# Assessment of Pulmonary Arterial Hypertension by Magnetic Resonance Imaging

**DOI:** 10.18383/j.tom.2015.00118

**Published:** 2015-09

**Authors:** El-Sayed H. Ibrahim, Abubakr A. Bajwa, Richard D. White

**Affiliations:** 1 Department of Radiology, University of Michigan, Ann Arbor, MI;; 2 Department of Medicine, University of Florida, Jacksonville, FL; and; 3 Department of Radiology, Ohio State University, Columbus, OH

**Keywords:** pulmonary arterial hypertension, magnetic resonance imaging, pulmonary artery, right ventricle

## Abstract

Pulmonary arterial hypertension (PAH) is characterized by elevated pulmonary artery pressure (PAP), altered pulmonary artery (PA) hemodynamics, and vessel wall characteristics that affect the right ventricular (RV) function. Magnetic resonance imaging (MRI) has recently been considered in PAH and has shown promising results for estimating PAP, measuring PA hemodynamic parameters, assessing PA vessel wall stiffness, and evaluating RV global and regional functions. In this article, we review various MRI techniques and image analysis methods for evaluating PAH, with an emphasis on the resulting images and how they are interpreted for both qualitatively and quantitatively assessing the PA and RV conditions.

## Introduction

Pulmonary arterial hypertension (PAH) is a progressive disorder characterized by elevated pulmonary artery pressure (PAP) and pulmonary artery (PA) vascular resistance, which increase the right ventricular (RV) afterload and lead to RV dysfunction and ultimately right-sided heart failure (1–3). Although PAP measurement by right heart catheterization is the current gold standard for confirming PAH, the technique's invasiveness precludes its use on a regular basis.

Although echocardiography is the modality of choice for evaluating PAH because of its bedside availability and relatively low cost, magnetic resonance imaging (MRI) has recently been considered as a potential “one-stop-shop” imaging modality in PAH (4–7), where different techniques have been developed for estimating PAP (8, 9) and deriving cardiovascular parameters for evaluating PA hemodynamics (10, 11), PA vessel wall characteristics (12), RV function (11, 13), and left ventricular (LV) involvement (14). Compared with the established echocardiography, MRI capabilities far exceed those of ultrasound without the geometric assumptions or acoustic window limitations. Furthermore, using advanced techniques (as will be illustrated in this article), MRI could be used to provide important parameters that reflect PA vessel wall stiffness, pulmonary pressure, and regional myocardial function. Nevertheless, our understanding of the interrelationship between PA hemodynamics and RV function during PAH development is still limited. In this article, we provide a pictorial review of various MRI techniques and analysis methods for evaluating PAH by qualitatively and quantitatively assessing the PA and RV conditions on patients with different disease stages.

## MRI Techniques

The imaging protocol described in this article has been optimized on a 3T MRI scanner (Siemens Medical Solutions, Erlangen, Germany); therefore, implementation on 1.5T scanners may need some parameter optimization. The imaging protocol lasts for approximately 30 min and includes the following sequences:

1. *Cine images for evaluating the heart morphology (chamber size and wall thickness), heart function (ejection fraction [EF]), myocardial trabeculation, and septal wall curvature.* A gradient-echo pulse sequence is typically used for cine imaging with the following parameters: slice thickness, 8 mm; spatial resolution, 2 mm; repetition time (TR), 43 ms; echo time (TE), 2.4 ms; number of averages, 1; percentage sampling, 75%; bandwidth (BW)/pixel, 473 Hz; and number of cardiac phases, 23.
2. *Tissue tagging or strain encoding for measuring myocardial strain and strain rate (15).* Typical imaging parameters for myocardial tagging are as follows: slice thickness, 8 mm; spatial resolution, 1.25 mm; TR, 91.7 ms; TE, 4 ms; number of averages, 1; percentage sampling, 75%; BW/pixel, 185 Hz; number of cardiac phases, 25; flip angle, 8°; and tag separation, 6 mm. Strain encoding (SENC) is a relatively new imaging technique that requires the availability of research patch or pulse sequence programming for the scanner to run the sequence. Compared to conventional tagging, SENC provides much higher-resolution strain maps (on the pixel level) with intuitive color-coded images.
3. *Tricuspid velocity-encoding flow images for evaluating diastolic function (early-to-atrial filling ratio [E/A]) and tricuspid regurgitation*. Typical imaging parameters for valvular flow imaging are as follows: slice thickness, 8 mm; spatial resolution, 2.8 mm; TR, 22 ms; TE, 2 ms; number of averages, 1; percentage sampling, 80%; BW/pixel, 1500 Hz; number of cardiac phases, 60; flip angle, 15°; and velocity-encoding setting (venc), 150 cm/s.
4. *PA velocity-encoding flow images for assessing PA distensibility (cross-sectional area change between end diastole and end systole), hemodynamic parameters (acceleration rate, ejection volume, and ejection time), and pulse wave velocity (ratio of flow change to area change at early systole) (16).* Velocity-encoding PA imaging parameters are as follows: slice thickness, 8 mm; spatial resolution, 0.7 mm; TR, 29 ms; TE, 4 ms; number of averages, 1; percentage sampling, 90%; BW/pixel, 337 Hz; number of cardiac phases, 80; flip angle, 15°; venc, 150 cm/s.

## MRI Quantitative and Qualitative Analysis

### RV Morphology and Function

**Figure 1.**
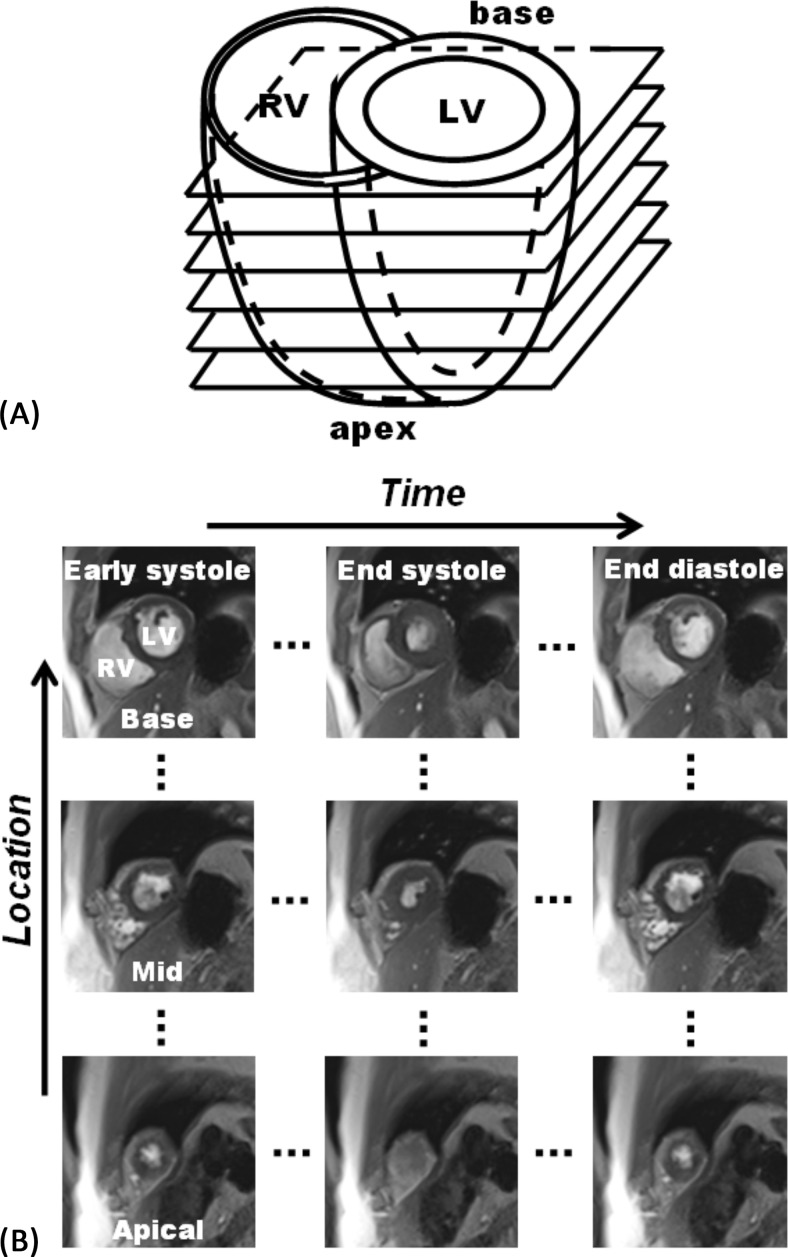
Cine MR images. (A) Stack of parallel short-axis images covering both the right and left ventricles. (B) Sample images at different locations in the heart and times in the cardiac cycle.

**Figure 2.**
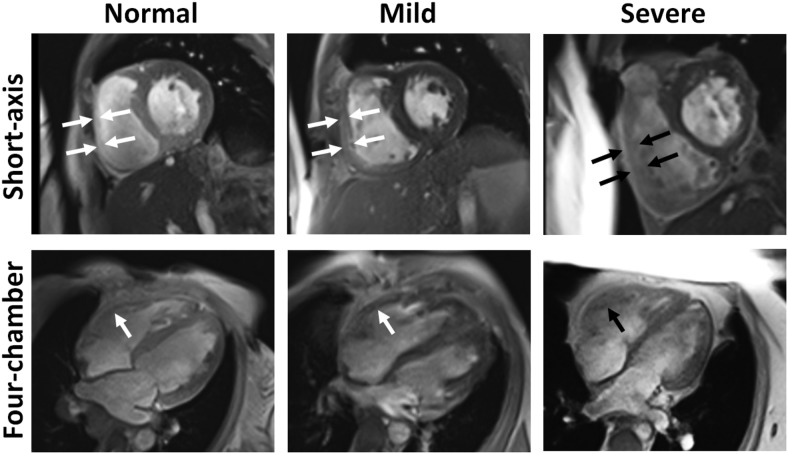
Right ventricular (RV) hypertrophy in pulmonary arterial hypertension shown on cine MR images. Short-axis (top) and the corresponding 4-chamber (bottom) slices of a patient without hypertrophy (left) and patients with mild (middle) and severe (right) RV hypertrophy. Note the thick RV wall, which is translated into increased RV mass.

**Figure 3.**
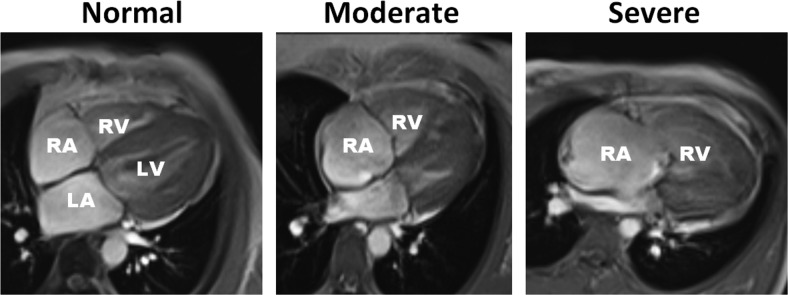
Right ventricular (RV) dilation. Four-chamber cine MR images showing a patient with nondilated RV (left) as well as patients with moderate (middle) and severe (right) RV dilation. Note the large chamber size, which leads to increased RV volume. The right atrium shows corresponding dilation in the provided examples.

**Figure 4.**
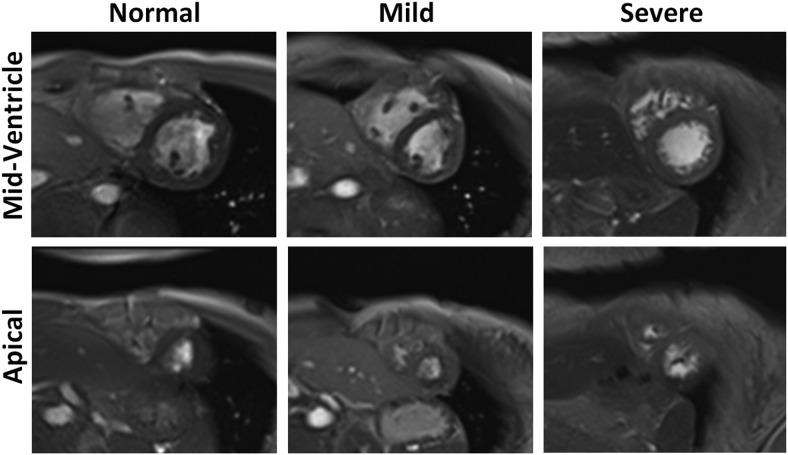
Myocardial trabeculations in the right ventricle, as shown on short-axis cine MR images. Trabeculations are especially apparent at the midventricular (top) and apical (bottom) slices. Examples of normal (left), mild PAH (middle), and severe PAH (right) cases are shown.

**Figure 5.**
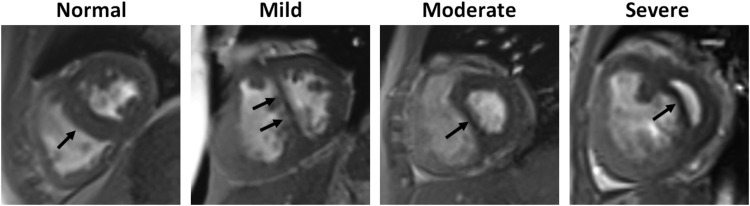
Septal wall bending in pulmonary arterial hypertension (PAH). Basal short-axis cine MR images at early diastole showing normal septal wall shape (left) as well as mild, moderate, and severe leftward septal wall bowing at different PAH stages.

Cardiac cine imaging provides a set of parallel short-axis (SAX) slices that cover the whole heart at consecutive frames throughout the cardiac cycle (Figure 1). Long-axis (LAX) four-chamber cine images are usually acquired as well. The LV endocardial and epicardial boundaries are delineated in the resulting stack of parallel SAX images, from which ventricular mass and volumes are calculated, and used to examine the existence of ventricular hypertrophy (Figure 2) or dilation (Figure 3), respectively. Dilation of the right atrium is not uncommon in PAH as well. Further examination of the SAX slices provides an evaluation of the degree of myocardial trabeculation (Figure 4). For quantitative results, the cine images are processed to measure RV EF. LV EF could also be computed to examine the degree of LV involvement (14). Normal values for different RV variables have been provided for males/females as follows (17): EF, 62±10 / 69±10%; SAX end-diastolic diameter, 35.3±6.8 / 31.6±1.7 mm; 4-chamber end-diastolic diameter, 39.1±5.2 / 36.5±1.2 mm; 4-chamber length, 72.9±8.8 / 67±2.9 mm; and right atrial diameter, 43±5.6 / 39.7±1.6 mm. Furthermore, in normal individuals, the RV wall is thin, and the RV mass is less than the LV mass. In severe PAH, the RV pressure far exceeds that of the LV, which results in leftward bending of the interventricular septal wall, compromising the LV cavity (Figure 5). The septal wall curvature is calculated as a measure of RV pressure (18).

### Myocardial Contractility

**Figure 6.**
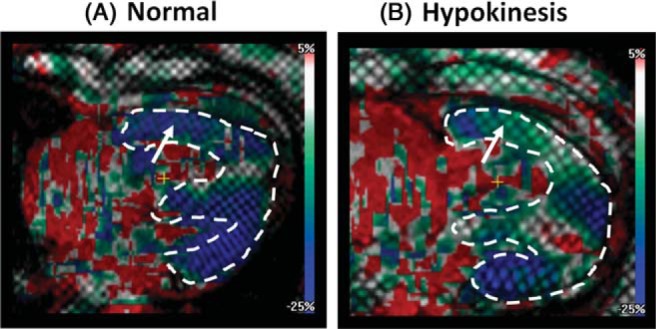
Right ventricular (RV) myocardial strain using myocardial tagging and harmonic phase analysis. Color-coded grid-tagged images showing longitudinal strain. The images show 2 examples of patients with normal RV contraction (A) and RV hypokinesis (B).

Despite the importance of global heart function measures (e.g., EF), no information about myocardial contractility could be obtained from the cine images. The importance of the regional heart function measures is that they provide an early warning of the heart dysfunction before global heart function measures are altered (15). Regional myocardial contractility measures are usually obtained from tagged MR images, in which patterns of saturated magnetization (usually parallel lines or grids) are superimposed on the myocardium, which becomes deformed during the cardiac cycle after the heart contracts. By analyzing the motion of these “tags,” myocardial strain and strain rate could be obtained (Figure 6). The tagged images are analyzed using commercial or custom-built software packages, where the operator selects a few points on the myocardium that are automatically tracked through the cardiac cycle to measure tissue motion and calculate mechanical parameters of myocardial deformation, including strain, strain rate, and torsion. It should be noted that circumferential and radial strains are measured from SAX images, whereas longitudinal strain is measured from LAX images. Of importance in PAH is measuring longitudinal strain, which is the main strain component in the RV. The percentage segmental shortening of the RV free wall increases gradually through systole to average values (across all segments) of 12%, 14%, and 16% at the basal, midventricular, and apical slices, respectively (19).

**Figure 7.**
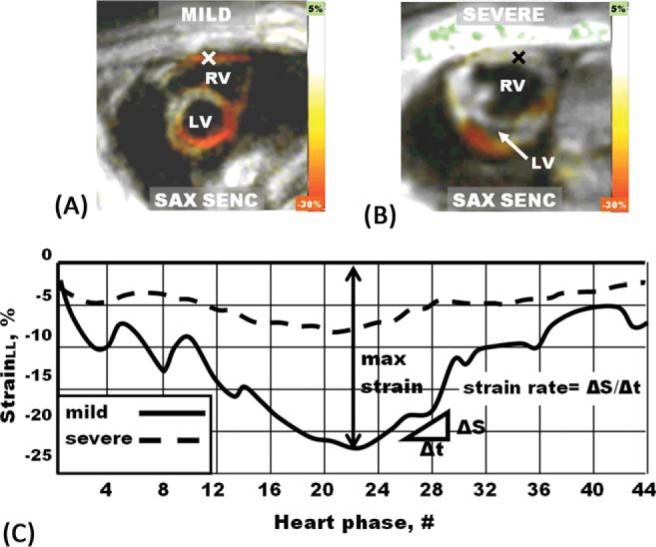
Strain encoding (SENC) MR images showing myocardial strain. Color-coded SENC images show through-plane longitudinal strain in patients with mild (A) and severe (B) pulmonary arterial hypertension. (C) Longitudinal right ventricular strain curves and early-diastolic strain rate at a remote RV site marked with ′x'.

SENC is a relatively new MRI technique for measuring myocardial through-plane strain, which is advantageous to conventional tagging because of its high-resolution, simple postprocessing, and intuitive color-coded strain presentation (15). These advantages make SENC especially suitable for measuring RV strain. In contrast to conventional tagging, SENC does not show any tagging pattern; rather, strain is directly represented in a color-coded strain map. Because SENC measures through-plane strain, longitudinal and circumferential strains are measured from SAX and LAX SENC images, respectively. Figure 7 shows SENC images and RV strain curves, where PAH is characterized by reduced strain. Despite the low resolution of the resulting images (as a result of the patients' limited breath-holding capabilities, the image was acquired in real time with scan time of only one heartbeat), the images clearly show compromised RV contractility compared to that of the LV. Strain rate is another important parameter that can be measured at any point in the cardiac cycle. PAH is associated with reduced early-diastolic strain rate, which reflects a compromised RV diastolic function, as explained further in the following section.

### Diastolic RV Function and the Tricuspid Valve

**Figure 8.**
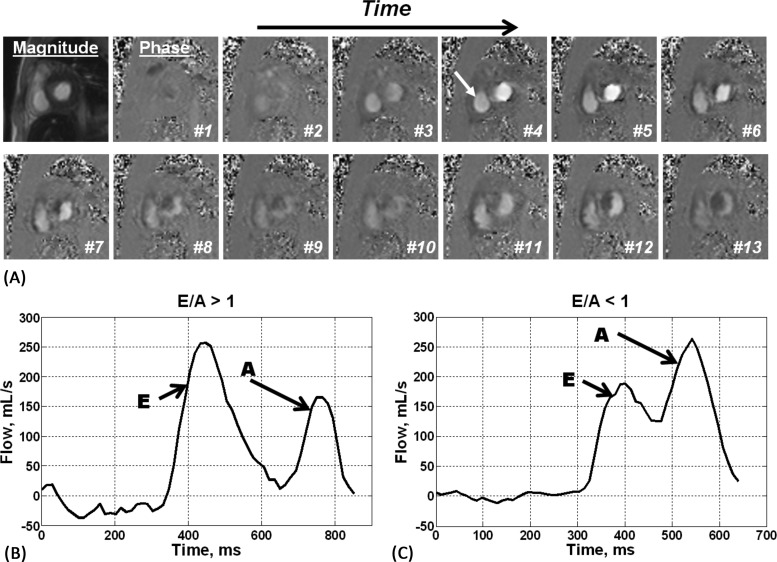
Blood flow through the tricuspid valve during diastole, as shown on phase-contrast MR images acquired across the tricuspid valve with through-plane velocity encoding. (A) Reference magnitude image and a sequence of phase images at consecutive timepoints in the cardiac cycle. The tricuspid valve (arrow) opens, and the blood flows from the right atrium to the right ventricle. (B, C) Tricuspid blood flow curves from 2 patients with an early-to-atrial filling ratio greater than 1 (B) and less than 1 (C), reflecting normal and abnormal diastolic functions, respectively.

The RV diastolic function could be evaluated by analyzing the blood flow pattern through the tricuspid valve. As shown in Figure 8, 2 sets of velocity-encoded magnitude and phase images are acquired at the tricuspid valve level. The images are used to derive the flow curve, from which the early and atrial filling peaks are identified. Using flow analysis software packages, which can be provided by the scanner manufacturer or by third-party software development companies, the operator semiautomatically delineates the valve contour in the acquired velocity-encoded images throughout the cardiac cycle. Typically, the operator needs only to identify the contour at 1 time frame, upon which the software propagates the selected region to other time frames throughout the cardiac cycle. Blood velocity and valve cross-sectional area are automatically determined from the magnitude and phase images, respectively, based on which flow is measured and various hemodynamic parameters are derived. The E/A diastolic filling ratio provides information about the diastolic function (20). Typically, an E/A ratios greater than 1 and less than 1 are associated with normal and abnormal functions, respectively. However, it should be noted that an E/A ratio greater than 1 could exist in pseudonormal or resistive diastolic dysfunctions as well. Another benefit of the flow curve is using the tricuspid regurgitation velocity for evaluating the RV pressure.

### PA Dilation

**Figure 9.**
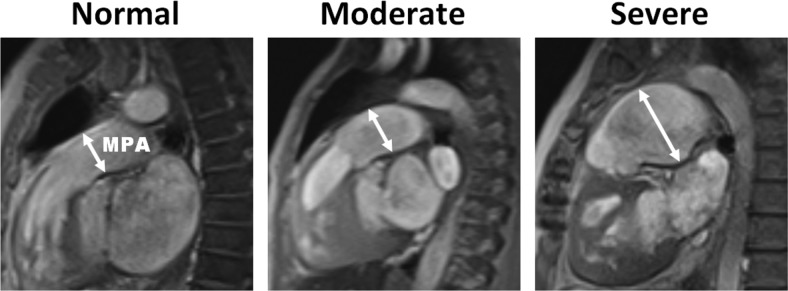
Pulmonary artery (PA) dilation, as shown on oblique sagittal anatomical images. Oblique images show a patient with normal PA size (left), as well as patients with moderate (middle) and severe (right) PA dilation. MPA, main PA.

The PA morphology and size can be assessed from oblique sagittal slices. In normal subjects, the PA cross-sectional area is smaller than that of the aorta. With elevated PAP in PAH, the PA dilates and could even become larger than the aorta, as shown in Figure 9. PA dilation affects its viscoelastic properties, which compromises the efficiency of the pulmonary circulation.

### PA Hemodynamics

**Figure 10.**
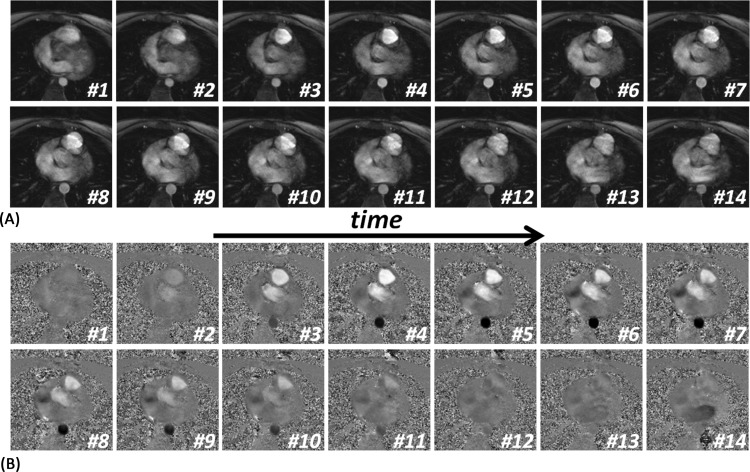
Consecutive frames of magnitude (top) and phase (bottom) velocity-encoded images acquired at the pulmonary valve level during systole. The images are used to draw the flow curve, from which pulmonary artery hemodynamic parameters are measured.

**Figure 11.**
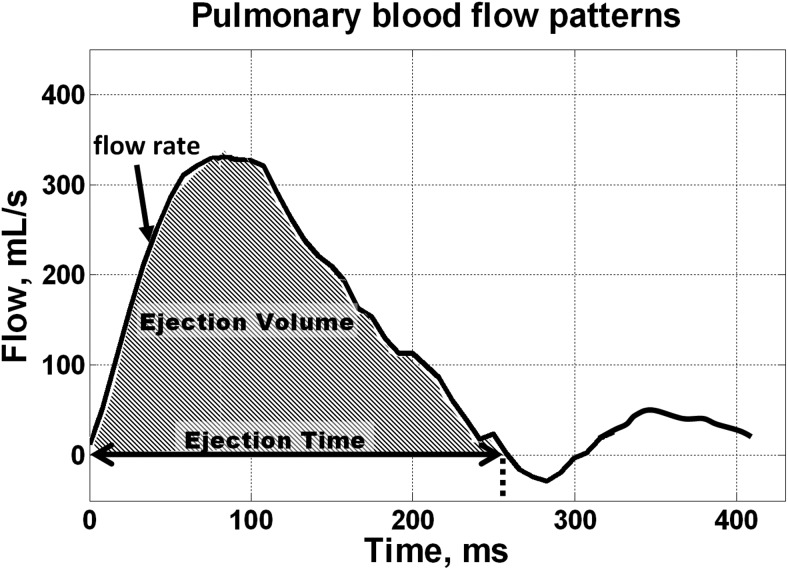
Pulmonary flow pattern and hemodynamic parameters based on velocity-encoding MRI. The flow curve and parameters are calculated from the phase-encoded images acquired perpendicular to the flow direction at the pulmonary valve level.

The PA condition could be evaluated by measuring various hemodynamic parameters derived from the flow images acquired at the pulmonary valve level. The imaging sequence results in 2 sets of velocity-encoding magnitude and phase images (10), as shown in Figure 10. The resulting images are analyzed to derive the flow curve (Figure 11), from which various hemodynamic parameters can be calculated (10). Analyzing the PA velocity-encoded images is conducted in a fashion similar to valvular flow analysis described previously. The information provided by velocity-encoding MRI far exceeds that provided by angiography. Not only does phase-encoding MRI provide information about the vascular structure, it also provides information about hemodynamics, including blood flow volume, flow rate, and velocity for comprehensively evaluating the pulmonary circulation. PAH has been associated with increased acceleration rate and reduced ejection time and ejection volume. Postsystolic retrograde flow is not uncommon in moderate-to-severe PAH.

### PA Vessel Wall Stiffness

**Figure 12.**
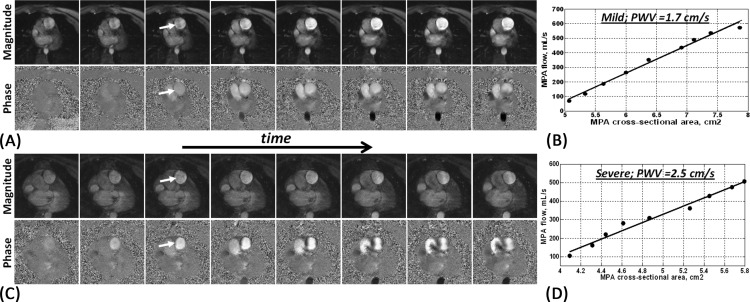
Pulmonary artery (PA) distensibility and pulse wave velocity (PWV), as measured using the flow-area method applied on velocity-encoded MRIs. (A) Successive frames of magnitude (top) and the corresponding phase (bottom) images showing early-systolic blood flow in the PA (arrow) in a patient with mild pulmonary arterial hypertension (PAH). Noticeable increases in the PA cross-sectional area and blood flow are observed. (B) PWV is calculated from the images in (A) as the ratio of change in blood flow to change in PA area. Images shown in (C) and (D) are similar to (A) and (B) in a patient with severe PAH (slight change in PA cross-sectional area, which results in a large PWV).

PA compliance and vessel wall stiffness could be assessed from distensibility and pulse wave velocity (PWV) measurements (16). Figure 12 shows velocity-encoding magnitude and phase images across the PA and the measured PWV in 2 individuals with mild and severe PAH. For measuring PWV, the PA cross-section has to be delineated at a few timeframes during systole, from which PA cross-sectional area and flow are determined and plotted against each other. PWV is calculated as the slope of the line fitted to the flow-area measurements at early systole. Normal PWV values in the left and right PA have been reported as 2.09 ± 0.64 m/s and 2.33 ± 0.44 m/s, respectively (21). PAH is associated with reduced vessel distensibility (minimal change in the PA cross-sectional area during systole) and increased PWV (fast pressure wave in the PA as a result of its stiff wall).

## Conclusions

In PAH, the elevated PAP alters the PA hemodynamic profile and results in dilated PA and increased vessel wall stiffness, which increase the RV afterload. The RV responds by building new sarcomeres in parallel (resulting in RV hypertrophy) and in series (resulting in RV dilation). In severe PAH, the elevated RV pressure causes a leftward septal wall bending. The undergoing ventricular remodeling compromises both the RV local and global functions, which leads to diastolic dysfunction and ultimately heart failure in severe PAH. Compared with current standard-of-care protocols, the different images resulting from the described MRI protocol provide detailed information about PAH in terms of PA compliance and RV function that shed more light on PAH pathophysiology. For example, measuring regional myocardial function provides information about early-disease development, and PWV and PA hemodynamics provide noninvasive surrogate markers about the right-sided heart pressure and vessel wall stiffness.

Despite the advantages provided by MRI, several points have to be taken into consideration to generate artifact-free images and to obtain accurate measurements. For example, sufficient spatial resolution has to be ensured to avoid measurements underestimation. Similarly, temporal resolution has to be high enough to generate accurate time-related parameters, e.g., strain rate, peak strain, and various hemodynamic parameters. In the case of velocity-encoding imaging, the venc parameter has to be set slightly higher than the maximum expected velocity to avoid aliasing artifacts while remain sensitive to detecting small velocity changes. Finally, small tag separation has to be used in myocardial tagging for effective regional functional analysis, especially in the RV.

In summary, MRI is expected to play a key role for better understanding the PAH pathophysiology by illustrating the relationships between various PA- and RV-related parameters and their relative importance at various disease stages. It is expected that adopting the provided imaging protocol in clinical practice would allow for better patient evaluation, prognosis, and treatment planning. For example, by identifying early markers of regional myocardial dysfunction, early treatment can be initiated before heart failure development. Furthermore, the degree of deviation of the MRI-derived parameters from normal ranges would help determine the appropriate treatment and level of intervention, if needed.

**Conflict of Interest:** The authors declare no conflicts of interest.
